# Improvement of the gait deviation index for spinal cord injury to broaden its applicability: the reduced gait deviation index for spinal cord injury (rSCI-GDI)

**DOI:** 10.3389/fbioe.2024.1431596

**Published:** 2024-10-02

**Authors:** Diana Herrera-Valenzuela, Isabel Sinovas-Alonso, Ana de los Reyes, Ángel Gil-Agudo, Antonio J. del-Ama

**Affiliations:** ^1^ International Doctoral School, Rey Juan Carlos University, Madrid, Spain; ^2^ Biomechanics and Technical Aids Unit, National Hospital for Paraplegics, Toledo, Spain; ^3^ Unit of Neurorehabilitation, Biomechanics and Sensorimotor Function (HNP-SESCAM), Associated Unit of R&D&I to the CSIC, Madrid, Spain; ^4^ Department of Applied Mathematics, School of Science and Technology, Materials Science and Engineering and Electronic Technology, Rey Juan Carlos University, Móstoles, Madrid, Spain

**Keywords:** gait deviation index (GDI), spinal cord injury (SCI), gait impairment, threedimensional (3D) kinematic gait data, singular value decomposition

## Abstract

**Background:**

The SCI-GDI is an accurate and effective metric to summarize gait kinematics in adults with SCI. It is usually computed with the information registered with a photogrammetry system because it requires accurate information of pelvic and hip movement in the three anatomic planes, which is hard to record with simpler systems. Additionally, due to being developed from the GDI, the SCI-GDI is built upon nine joint movements selected for a pediatric population with cerebral palsy, for which the GDI was originally developed, but those nine movements are not necessarily as meaningful for adults with SCI. Nevertheless, pelvic movement and hip rotation have been proven to have low reliability even when acquired with gold-standard photogrammetry systems. Additionally, the use of photogrammetry is limited in real-life scenarios and when used with rehabilitation technologies, which limits the use of the SCI-GDI to evaluate gait in alternative scenarios to gait laboratories and to evaluate technologies for gait assistance. This research aimed to improve the SCI-GDI to broaden its applicability beyond the use of photogrammetry.

**Methods:**

An exploration of the mathematical relevance of each joint movement included in the original GDI for the performance of the metric is performed. Considering the results obtained and the clinical relevance of each of the 9 joints used to compute the SCI-GDI in the gait pattern of the SCI population, a more adaptable SCI-GDI is proposed using four joint movements that can be precisely captured with simpler systems than photogrammetry: sagittal planes of hip, knee and ankle and hip abduction/adduction.

**Results:**

The reduced SCI-GDI (rSCI-GDI) effectively represents gait variability of adults with SCI as does the SCI-GDI, while providing more generalizable results and equivalent or stronger correlations with clinical tests validated in the population. During the derivation of the improved index, it was demonstrated that pelvic movements, hip rotation, and foot progression angle introduce high variability to the dataset of gait patterns of the adult population with SCI, but they have low relevance to characterize gait kinematics of this population. The rSCI-GDI can be calculated using the 14-feature vectorial basis included in the electronic addendum provided.

## 1 Introduction

The SCI-GDI (Gait deviation index for Spinal cord injury) is an accurate and effective metric to summarize gait kinematics in adults with SCI. The GDI and the SCI-GDI are usually computed with information retrieved from a 3DGA (three-dimensional gait analysis) performed using a photogrammetry system. Since it requires accurate pelvic and hip movement information in the three anatomic planes, it is hard to compute it with data recorded with simpler systems. Additionally, due to being developed from the GDI, the SCI-GDI is built upon nine joint movements selected for a pediatric population with cerebral palsy, for which the GDI was originally developed (Schwartz and Rozumalski, 2008), but those nine movements are not necessarily as meaningful for adults with SCI. These are important limitations for various reasons. Firstly, pelvic movement has been proven to have low reliability even with gold-standard photogrammetry systems due to anatomic constraints for accurately marking the ideal anatomical landmarks (Herrera-Valenzuela et al., 2022; O’Sullivan et al., 2010; Langley et al., 2019). Similarly, hip rotation in the transversal plane has been shown to have low reliability even when acquired with photogrammetry systems (Baker et al., 2012). These are consequences of the intra and inter-rater variability generated because marker positioning depends on the expertise and perception of the examiner (Fonseca et al., 2023). Additionally, the use of photogrammetry is limited in real-life scenarios because it requires a constrained scenario to work properly This limits the use of the SCI-GDI to evaluate gait during activities of daily living (ADL) in alternative scenarios to gait laboratories. Besides, the instrumentation required for photogrammetry turns complicated to implement when used with rehabilitation technologies, limiting the possibility of using the SCI-GDI to evaluate technologies for gait assistance due to the likelihood of having a high rate of marker occlusion and the need to adapt the models to compute kinematics, which reduce its accuracy.

In consequence, this research aimed to improve the SCI-GDI to broaden its applicability beyond the use of photogrammetry. To this end, an adapted version of the SCI-GDI, including kinematics of fewer joints movements than the ones used to compute it, was developed. The same dataset used in the derivation of the SCI-GDI (Herrera-Valenzuela et al., 2022) was used in this exploration to compare the effects of reducing the input kinematics of the index in the same sample of adults with iSCI (incomplete spinal cord injury). Priority was given to the most clinically relevant joint movements for the population with SCI that could be acquired with precision with simpler and more versatile systems than photogrammetry (ElineNijmeijer et al., 2023; Blanco-Coloma, 2023).

This article explores mathematically the relevance of each joint movement included in the original GDI for the performance of the metric. Considering these results, in addition to the clinical relevance of each of the 9 joints used to compute the SCI-GDI in the gait pattern of the iSCI population, a more adaptable SCI-GDI is proposed using fewer joint movements that can be precisely captured with simpler systems than photogrammetry. This new index is the reduced SCI-GDI (rSCI-GDI). The metric is compared with the GDI-SCI to assess their differences and is validated against other validated clinically meaningful scales used in the population with SCI.

## 2 Materials and methods

### 2.1 Dataset

The same datasets used for the derivation of the SCI-GDI were used in this study (Herrera-Valenzuela et al., 2022). It contains the kinematic data from 3DGA of 302 strides from patients with a diagnosis of SCI aged between 16 and 70 years old (y.o.) (33.91 ± 17.86), with neurological levels of injury between C1 and L5 and ASIA (American Spinal Injury Association) impairment scale (AIS) C-D, regardless of the etiology and time since injury. The detailed demographic and clinical characteristics of the sample are presented in Table 1. The control group comprises kinematic gait data of 446 strides from adults without gait impairment (HV) between 18 and 63 y.o. (35.10 ± 15.41). Both populations were registered at the Biomechanics and Technical Aids Unit of the National Hospital for Paraplegics in Toledo, Spain, using the same protocol for 3DGA.

**TABLE 1:** Demographic and clinical characteristics of the samples in the train and validation datasets.

Characteristic	Type	Train (n = 302)	Validation (n = 72)
Age	16–25	156	52
	26–40	32	0
	41–60	79	10
	>60	35	10
AIS	A	0	10
	C	36	10
	D	256	36
	Cauda equina	10	10
	N.A. (Congenital)	0	6
Time since injury	6 months (incl.) or less	58	10
	6 months (excl.) to 1 year (incl.)	40	0
	1 (excl.) to 5 years (incl.)	86	26
	More than 5 years	92	30
	Congenital	26	6
Injury level	C1-C8	153	0
	T1-T6	12	26
	T7-T12	68	20
	L1-L5	69	20
	N.A. (Congenital)	0	6
WISCI II level	12	2	0
	13	6	0
	15	18	10
	16	65	26
	18	12	6
	19	87	0
	20	112	30

All patients and HV signed an informed consent to perform the gait analysis. The study protocol was approved by the local bioethics committee (Clinical Research Ethics Committee at Complejo Hospitalario Universitario de Toledo, no. 823) and conformed to the Declaration of Helsinki.

### 2.2 Data analysis

All data analysis was performed with Matlab R2019a (The MathWorks, Inc., Natick, Massachusetts, United states).

#### 2.2.1 Mathematical exploration of the relevance of the 9 joint movements used in the GDI-SCI

The complete dataset with nine kinematic curves was modified by removing the three pelvic curves from the 302 strides. Afterwards, a leave-one-out experiment with the remaining six kinematic curves (three planes for the hip, knee flexion and ankle dorsi/plantarflexion and foot progression angle) was performed to identify the joints that introduce more variability of the dataset (i.e., the ones that, when knocked out, allow to successfully represent the dataset with an orthonormal basis of lower order). Each joint curve was removed from the dataset before computing the reduced order (*m*th order) orthonormal basis. A grid search considering values of *m* between 15 and 35 was used*.* In each case, we analyzed the order of the basis required to fulfill the three criteria required to select the least possible features to effectively represent the variability of the dataset and to allow high fidelity reconstructions, considered in the derivation of the original SCI-GDI (Herrera-Valenzuela et al., 2022). These are to account for at least 98.0% of the variance of the dataset (VAF≥98.0%), allowing to obtain a mean accuracy of 98% of the *m*th order reconstructed curves, and reconstructing most of the vectors dataset with fidelity ≥95.0%.

#### 2.2.2 Computation of the reduced SCI-GDI basis

Based on the results obtained in Section 2.2.1 in combination with the clinical experience and scientific evidence regarding the accuracy of the register of specific joint movements during 3DGA (Fonseca et al., 2023), a reduced GDI-SCI comprising only hip flexion/extension, hip abduction/adduction, knee flexion/extension and ankle dorsi/plantarflexion was computed and evaluated. Compared to the 9 joints used in the complete SCI-GDI, pelvic movements were removed due to the low reliability in capturing them even with photogrammetry systems due to anatomic constraints for accurately mark the ideal anatomical landmarks (Herrera-Valenzuela et al., 2022; O’Sullivan et al., 2010; Langley et al., 2019; Fonseca et al., 2023). Similarly, hip rotation was removed due to the poor inter-evaluator and moderate inter-trial and intra-evaluator reliability reported in 3DGA (Fonseca et al., 2023). Lastly, foot progression angle presents moderate reliability for these three aspects (Fonseca et al., 2023) but was removed mainly because it has little clinical relevance in SCI compared to ankle movements in the sagittal plane.

A matrix to compute the reduced order optimal basis was formed with the 302 strides from iSCI patients. This data is named as train dataset. We performed a grid search considering values of *m* between 10 and 25 to find the minimum features needed to form the optimal reduced order SCI basis with the three criteria explained at the end of section 2.2.1.

Furthermore, to validate the generalizability of the new basis built from iSCI gait data, a validation set was built with 72 additional strides that were not used to calculate the basis. These were reconstructed and compared using the SCI-GDI and the rSCI-GDI, and the reconstruction fidelity was assessed with the same criteria used in the train set, allowing to compare the quality of the reconstructions in foreign data.

#### 2.2.3 Comparison between the SCI-GDI and the rSCI-GDI with respect to the WISCI II scale

The rSCI-GDI was calculated for each stride of the dataset using the control group data described in section 2.1. Each gait analysis study had an associated WISCI II level according to the walking impairment of the patient when recording the study. rSCI-GDI data was grouped according to the corresponding WISCI II level and HV data was considered as an additional set. The dataset included WISCI II levels 12, 13, 15, 16, 18, 19, and 20. Normal distribution for each group was assessed with Kolmogorov-Smirnov tests (*p* < 0.05).

To facilitate the analysis, a histogram of the rSCI-GDI data comprised within each WISCI II level was calculated with a normal distribution curve fitted to its mean and standard deviation. A stratified result of the histograms was expected, in accordance with the ordinal nature of the WISCI II scale. Afterwards, one-way ANOVA tests were performed between the rSCI-GDI values of each pair of WISCI II levels to identify differences among groups (*p* < 0.05).

To seek differences between the original SCI-GDI (Herrera-Valenzuela et al., 2022) and the rSCI-GDI, both indexes were calculated for each stride of the dataset using the HV data gathered in our institution. Consequently, one-way ANOVA tests were performed between each pair of equivalent WISCI II levels to identify differences among groups (*p* < 0.05).

Additionally, to study the relationship between both indexes, Pearson’s correlation and linear regression were calculated between both GDI values using the whole dataset.

#### 2.2.4 Validation of the rSCI-GDI with respect to other clinical measures validated for the population with SCI

Gait improvement in SCI following rehabilitation is assessed using different procedures, metrics, and tools. 3DGA is the most comprehensive and precise technology to analyze gait that allows to objectively assess lower limb kinematics and kinetics, thus providing a powerful tool for quantifying gait impairment and, therefore, to assist decision-making for clinicians (Patrick, 2003; Sinovas-Alonso et al., 2021; Murphy et al., 2019; Baker et al., 2016). On the other hand, there are validated clinical tests to assess overall gait function; these can be categorical, like the Walking Index for Spinal Cord Injury (WISCI) (Dittuno et al., 2001); spatiotemporal walking-related, such as the 10-m walk test (10MWT) (Van Hedel et al., 2008), and the 6-min walking test (6MWT) (Brooks et al., 2003); and to assess balance, in the case of the timed up and go test (TUGT) (Podsiadlo and Richardson, 1991) and the Berg balance scale (BBS) (Berg, 1989), among others (Wyndaele and Wyndaele, 2006). Besides, tests of motor function and spasticity assessment are often performed, such as the Lower Extremity Motor Score (LEMS) and the Modified Ashworth Scale (MAS).

The construct validity of the rSCI-GDI was evaluated with a set of representative outcome measures to assess gait and balance. To this end, the same dataset used in the equivalent study done for the SCI-GDI was used (Sinovas-Alonso et al., 2023). It contains data from 35 adults with a diagnosis of SCI. The data was collected at the Biomechanics Unit of the National Hospital for Paraplegics (HNP), in Toledo, Spain. Besides the data collected using the standard protocol for 3DGA of the center, the 10MWT in both self-selected and maximum speeds, the TUGT, and the LEMS were gathered during the sessions. LEMS assessment comprised five key muscles of each lower limb: hip flexors, knee extensors, ankle dorsiflexors, long toe extensors, and ankle plantar flexors. The 10MWT and TUGT were recorded three times before the 3GDA and averaged for each subject. With the data collected, cadence, gait speed, stance percentage, step width, stride and step length, and the rGDI-SCI were calculated. The Spearman correlation coefficient between the rGDI-SCI and all the tests and spatiotemporal parameters was also computed. The normal distribution of all variables was evaluated with a Kolmogorov-Smirnov (KS) test. Descriptive statistics for each of these scales and the subjects’ demographics are summarized in Table 2.

**TABLE 2:** Demographic and clinical characteristics of subjects in the dataset.

Characteristic	Type	Number of subjects (n = 35)
Age	16–25	16
	26–40	4
	41–60	11
	>60	4
AIS	C	4
	D	29
	E	2
Etiology	Traumatic	17
	Non-traumatic	18
Time since injury	6 months (incl.) or less	16
	6 months (excl.) to 1 year (incl.)	2
	1 (excl.) to 5 years (incl.)	8
	More than 5 years	8
	Congenital	1
Injury level	C1-C8	9
	T1-T6	6
	T7-T12	10
	L1-L5	10
WISCI II level	12	3
	13	1
	15	4
	16	9
	18	2
	19	3
	20	13
TUGT	Mean ± STD	12.01 ± 4.89
	Min – Max (Q1-Q3)	5.61–23.23 (8.38–14.66)
10MWT	Mean ± STD (Self-selected speed)	12.32 ± 4.44
	Min – Max (IQR) (Self-selected speed)	6.48–23.15 (8.11–14–63)
	Mean ± STD (Max. speed)	9.63 ± 3.77
	Min – Max (Q1-Q3) (Max. speed)	4.47–19.61 (5.98–11.76)
LEMS	Mean ± STD	37.20 ± 7.71
	Min – Max (Q1-Q3)	18.00–48.00 (33.00–42.50)
GDI-SCI	Mean ± STD	70.49 ± 14.58
	Min – Max (Q1-Q3)	36.33–104.39 (62.66–77.99)

## 3 Results

### 3.1 Mathematical exploration of the relevance of the 9 joint movements used in the GDI-SCI

**FIGURE 1:**
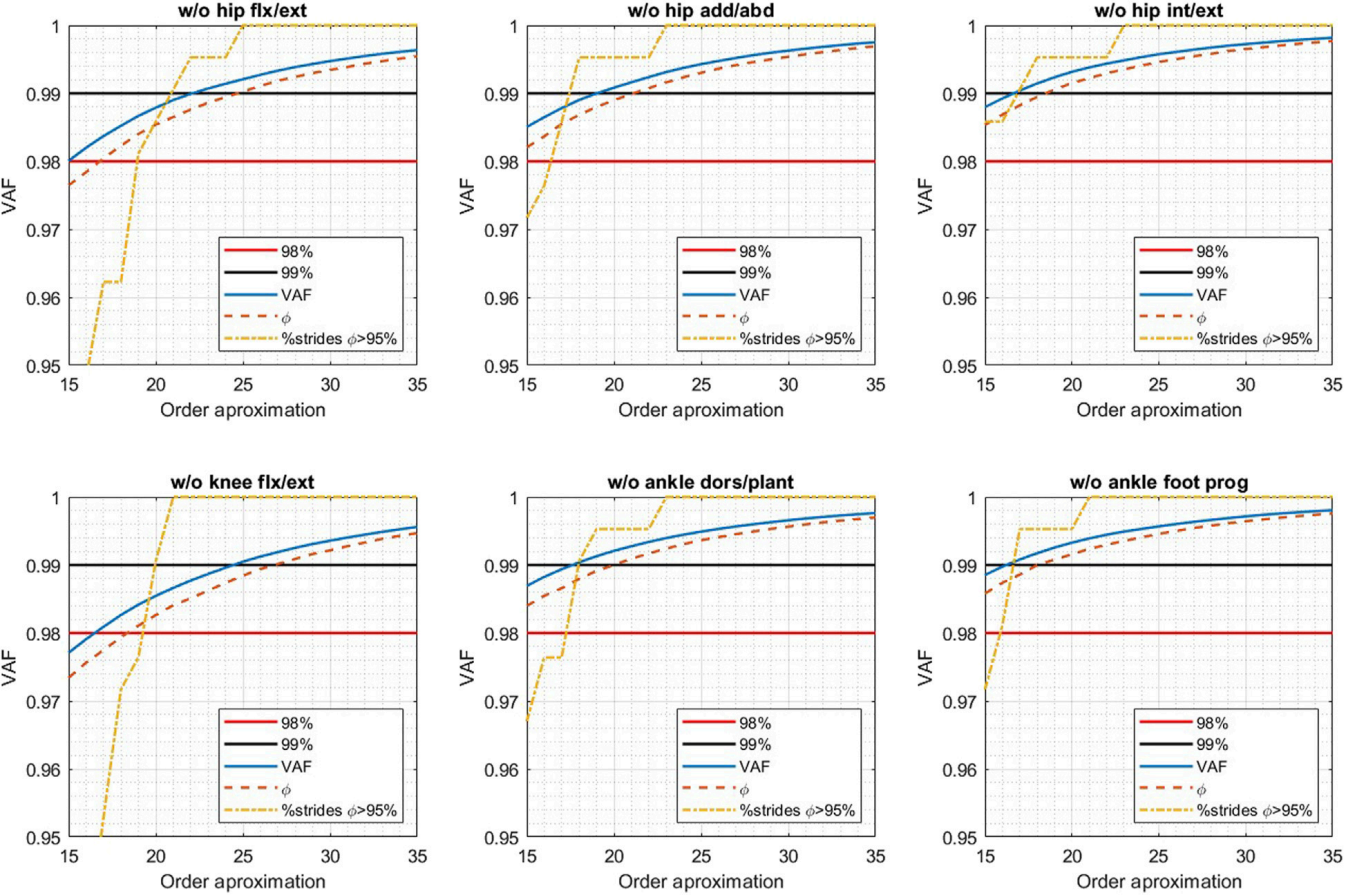
Results of the grid search exploration to find the reduced order basis required to fulfill the criteria required to have quality reconstructions when leaving out each one of the six joints of the dataset. For each order approximation, the blue lines indicate the VAF, the orange dotted line the average fidelity of the reconstructions and the yellow dotted line the percentage of the dataset reconstructed with fidelity over 95%. The red line indicates the threshold for the VAF, stated at 98%. The black line indicates the threshold of the percentage of the dataset reconstructed with fidelity over 95%, reported in 99% in the article of the original derivation of SCI (Schwartz and Rozumalski, 2008).

Results of the leave-one-out experiment are shown in Figure 1. The dataset is successfully represented requiring a lower order basis when removing hip internal/external rotation or the ankle foot progression angle.

### 3.2 Computation of the reduced SCI-GDI basis

**FIGURE 2:**
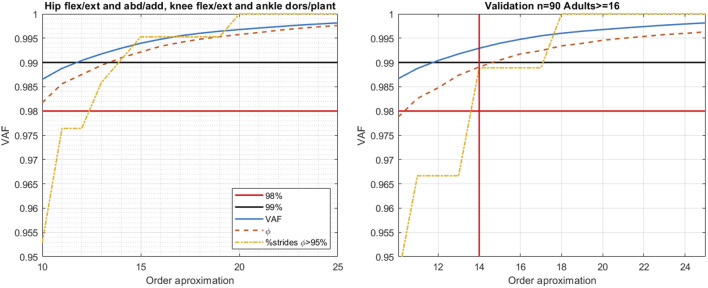
Results of the grid search exploration with the 4-joint dataset. The first 14 components of the reduced order basis allow to fulfill the criteria required to have quality reconstructions in both the train (left) and validation (right) datasets. For each order approximation, the blue lines indicate the VAF, the orange dotted line the average fidelity of the reconstructions and the yellow dotted line the percentage of the dataset reconstructed with fidelity over 95%. The red line indicates the threshold for the VAF, stated at 98%. The black line indicates the threshold of the percentage of the dataset reconstructed with fidelity over 95%, reported in 99% in the article of the original derivation of SCI (Schwartz and Rozumalski, 2008).

Figure 2 contains the results obtained in both train and validation datasets with the 4-joint reduced SCI-GDI basis. Results in both the train and validation set show that 14 features are enough to fulfill the three criteria considered for the creation of the GDI. In consequence, the orthonormal basis for the reduced SCI-GDI is built with the first 14 features of the basis built comprising kinematic data of only hip flexion/extension, hip abduction/adduction, knee flexion/extension and ankle dorsi/plantarflexion.

**FIGURE 3:**
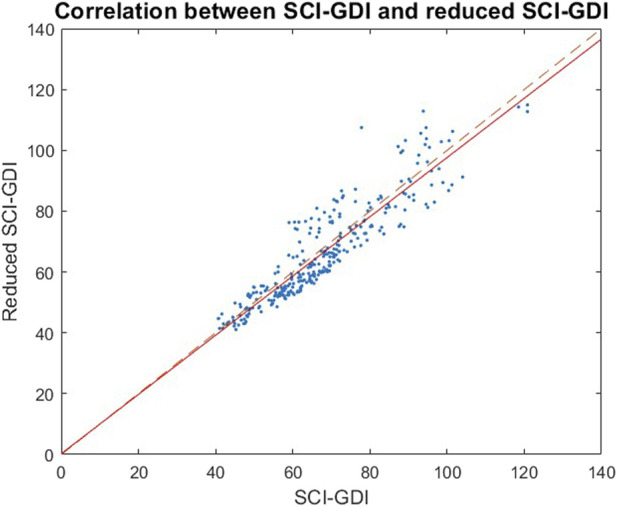
A strong linear correlation between the SCI-GDI and the reduced SCI-GDI was found (*r* = 0.9118). The linear regression between both indexes is represented by the continuous line, whereas the dashed line indicates the 1:1 axis. For less impaired subjects, lower reduced SCI-GDI can be assigned with respect to SCI-GDI values. The difference between both indexes is larger in data with less impairment and it reduces progressively towards more impaired gait patterns.

The summary of the results obtained for each criterion with this 14-feature reduced SCI-GDI basis in comparison to the ones obtained with the 21-feature SCI-GDI basis are summarized in 3. The best results in terms of average fidelity of the reconstructions and percentage of vectors reconstructed with a fidelity ≥95% were obtained with our *m* = 21 basis. In the validation set, equivalent results to those obtained in the train dataset were obtained for all criteria. A strong correlation between both indexes was found (r = 0.9118) and can be seen in Figure 3.

### 3.3 Comparison between the SCI-GDI and the rSCI-GDI with respect to the WISCI II scale

**FIGURE 4:**
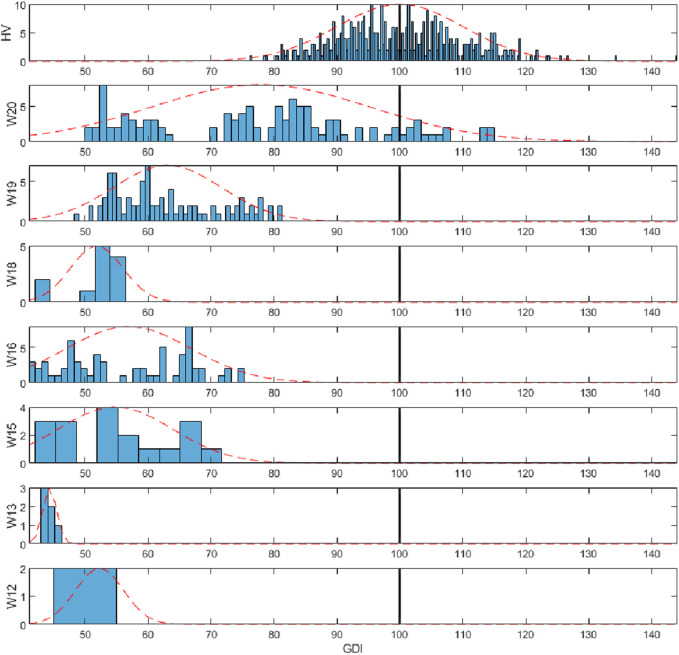
Histograms of the reduced SCI-GDI stratified by WISCI II level (12–20 and healthy volunteers). The dotted line represents the normal distribution curve fitted to the data within each level. The vertical black line indicates the control mean.

The results showed that the SCI-GDI is normally distributed across all WISCI II levels and in the HV group. The stratification of the reduced SCI-GDI with respect to the WISCI II levels comprised in the dataset used was confirmed, except for levels 18 and 13 (see Figure 4). The sensitivity of the reduced SCI-GDI with respect to WISCI II levels is limited. Statistically significant differences were found between all levels but between level 19 and level 12; between level 18 and levels 12, 15 and 16; between level 16 and levels 18, 15 and 12; and between level 15 and levels 18, 16 and 12. The only difference in the sensibility of both indexes is that the SCI-GDI can differentiate WISCI levels 15 and 16, unlike the reduced SCI-GDI. Additionally, statistically significant differences were only found between both indexes for the data of the WISCI level 19 (*p* = 0.0036).

### 3.4 Validation of the rSCI-GDI with respect to other clinical measures validated for the population with SCI

The rSCI-GDI presents very strong correlation with the SCI-GDI (r = 0.901), with a similar coefficient to the one obtained with the dataset used for the derivation of the index (r = 0.912). Moderate correlations were found between the index and the LEMS (r = 0.612), TUGT (r = −0.669), the 10MWT for both self-selected (r = −0.769) and maximum speed (r = −0.791), cadence (r = 0.611), walking speed (r = 0.790), stance percentage (r = −0.684), and stride (r = 0.749) and step length (r = 0.760). Fair correlations were found with the WISCI II scale (r = 0.566) and step width (r = −0.373). The rSCI-GDI presents equivalent or stronger correlation coefficients with most of the clinical tests evaluated when compared to the SCI-GDI. Correlations with the TUGT, cadence, and stance percentage improve from fair to moderate, whereas the correlation with step width improves from poor to fair. Only the correlation with the LEMS decreases with the rSCI-GDI. The interpretation of the strength of the coefficients follow the guidelines in (Akoglu, 2018). The full set of correlations is presented in Table 3.

**TABLE 3:** Spearman correlation coefficients of the GDI-SCI and the rGDI-SCI with spatiotemporal features of gait and clinical tests validated in SCI. Correlation strength is classified following the guidelines in (Wyndaele and Wyndaele, 2006): very strong ≥0.8 (green), moderate ≥0.6 (light green), fair ≥0.3, and poor <0.3.

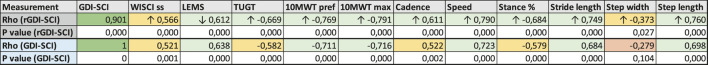

## 4 Discussion

The main objective of this article was to improve the SCI-GDI to broaden its applicability beyond the use of photogrammetry. To this extent, we derived and validated the reduced SCI-GDI. This study demonstrates that the rSCI-GDI effectively represents the variability of gait patterns among the population with iSCI, provides more generalizable results than the SCI-GDI and has equivalent or better correlations with clinical tests validated in the population. The rSCI-GDI is computed with a 14th order orthonormal basis derived from a dataset with four joint movements: hip flexion/extension, hip abduction/adduction, knee flexion/extension and ankle dorsi/plantarflexion.

The dataset of gait kinematics of adult population with iSCI is successfully represented requiring a lower order basis when removing hip internal/external rotation or the ankle foot progression angle (see Figure 1), indicating that these kinematics are the ones that introduce most variability to the dataset. Hip rotation in the transversal plane has been shown to have low reliability even when acquired with photogrammetry systems (Baker et al., 2012). Thereby, the variability introduced to the dataset of adult population with iSCI is likely not due to intrinsic gait characteristics of this neurological population but likely due to the intrinsic limitations of the acquisition system. As a consequence, this plane was removed from the ones used in the rSCI-GDI. Similarly, foot progression angle is the second joint with more variability when computing the Gait Variable Score (GVS) (Baker et al., 2012), a prior of the GDI. Moreover, this movement was included considering the population for which the GDI was originally computed (Schwartz and Rozumalski, 2008), but it has not been described as a relevant joint movement in the gait kinematics of people with SCI. As a consequence, supported in the clinical knowledge, in the results of the mathematical exploration of the impact of removing these joints, and in the technical viability for measuring each joint movement with commonly used systems, we computed a reduced SCI-GDI using only the movements of hip flexion/extension, hip abduction/adduction, knee flexion/extension and ankle dorsi/plantarflexion.

The rGDI-SCI shows a slightly better performance than the GDI-SCI in their respective training sets (containing the same subjects) for the three criteria evaluated: variance accounted for, similar average fidelity of reconstruction and similar percentage of gait vectors reconstructed with average fidelity ≥95% (see Table 4). Moreover, the rGDI-SCI shows better performance in the validation set and a negligible difference in the three measures between both sets, indicating that it is a more robust index than the SCI-GDI, with higher generalizability. These findings demonstrate that kinematics of pelvic movements in the three planes, hip rotation in the transversal plane, and the ankle foot progression angle, increment the variability of the gait kinematics within the adult population with iSCI due to difficulties in accurately measuring them, introducing noise in the captured data. When removed, consistent kinematic patterns of individuals with iSCI can be reconstructed with more precision, demonstrating that the remaining joint kinematics included in the calculation of the GDI (i.e., ankle, knee and hip flexion/extension and hip abduction/adduction) are more representative of this population. Strong evidence to support this fact is the almost equivalent performance of the rSCI-GDI in the validation dataset compared to the train dataset, because it demonstrates that the orthonormal basis derived from the reduced dataset allows to recover with high precision gait kinematics from foreign data. Unlike the SCI-GDI, whose orthonormal basis reconstructs less than 73% of the validation vectors with high fidelity (≥95%), more than 98% of the validation vectors of the reduced data fulfill the same criterion. The reduction from 21 to 14 eigenvectors to form the reduced order orthonormal basis from the SCI-GDI and the rSCI-GDI (respectively), could be explained because the less joint movements included, the less variability must be covered in the projections of the vectorial space covered by the orthonormal basis.

**TABLE 4:** Comparison of the quality of reconstruction of the whole dataset when using the reduced SCI-GDI basis with *m* = 14 and the SCI-GDI basis with *m* = 21 (Herrera-Valenzuela et al., 2023). Better results are obtained with the reduced SCI-GDI basis in the train and validation sets.

Basis (n^o^ of features)	Set	VAF (%)	Average fidelity of reconstruction (%)	% Of gait vectors reconstructed with average fidelity ≥95 (%)
GDI-SCI basis (*m* = 21)	Train	98.27	97.99	97.86
	Validation		94.74	72.22
Reduced GDI-SCI basis (*m* = 14)	Train	99.29	99.09	99.06
	Validation		98.91	98.89

VAF, Variance accounted for; SCI, Spinal cord injury; CP, Cerebral palsy; N/A, Not applicable.

Both indexes have a strong linear correlation (r = 0.9118), indicating they are effectively measuring similar aspects of gait of the SCI population. Bigger differences between both indexes can be observed in subjects with little gait impairment (see Figure 3), but statistically significant differences between both indexes were only observed in subjects in WISCI II level 19. Additionally, the only difference when assessing the sensibility of these indexes with respect to the WISCI II levels is that the GDI-SCI is sensible enough to differentiate levels 15 and 16, unlike the reduced version of the metric. This could be explained because the joint movements removed from the index (pelvic tilt, obliquity and rotation, hip rotation, and foot progression angle) have smaller angular variations between different functional levels (from 0.4° to 1.2°) compared to the variations of the remaining joints that are included in the rSCI-GDI (from 0.6° to 3.4°) (Baker et al., 2012). Therefore, while the reduced index manages to have enough sensibility to detect movements showing bigger differences, the reduction in joint movements used as an input compromise the index ability to detect the smaller differences of the removed joints that are related to the functionality of gait described by the WISCI II. In this regard, finding a limited relationship of the index with the WISCI II is expected due to the contrasting aspects of gait that they describe (Sinovas-Alonso et al., 2022). While the GDI describes gait kinematics, the WISCI II describes the ability to perform independent gait, measured by the number and type of technical aids and human support required to walk, which can be acquired with alternative gait patterns than the ones described by healthy controls.

Instead, the results of the validation of the rSCI-GDI against a broader set of clinically validated tests and spatiotemporal features of gait demonstrate the advantages of this reduced index with respect to the SCI-GDI (Sinovas-Alonso et al., 2023). The generalizability of the rSCI-GDI is confirmed by the very strong correlation found with the SCI-GDI calculated in this dataset, which was not used during the derivation of the reduced index. All correlations with the clinical scales are higher with the rSCI-GDI, being the only exception the LEMS, whose correlation decreased. Nonetheless, most of them remain in the same ranges of correlation strength. Interestingly, correlations with the TUGT, cadence, and stance percentage improve from fair to moderate; furthermore, the correlation with step width improves from poor to fair. Among these, the TUGT, the stance percentage and the step width are related to dynamic balance (Wellmon, 2007), indicating that although kinematics of the pelvic movement, hip rotation and foot progression are removed, the reduced index successfully conveys information related to the displacement and projection of the center of mass within the base of support, determinant of dynamic balance. The non-significant reduction in the correlation with the LEMS can be explained because this motor score evaluates hip flexors and knee extensors–including *rectus femoris*–, which are related to pelvic movement (Takahashi et al., 2021), thus, probably the SCI-GDI correlates better with the LEMS because it includes pelvic movement, unlike the rSCI-GDI.

The advantages of the rSCI-GDI with respect to the SCI-GDI are demonstrated in this this paper. It provides more generalizable results with higher quality reconstructions in foreign data, correlates better with most of the validated clinical scales in SCI and requires kinematic information of fewer joint movements to be computed. Thus, the use of this index is recommended to evaluate the gait of any person with an iSCI who walks independently regardless of the severity or neurological level of injury, from 16 to 70 years old in both men and women.

Despite being developed to be feasible to compute using the kinematics registered with simpler systems than photogrammetry, it is necessary to develop future studies that assess the concurrent validity of computing the rSCI-GDI with photogrammetry and with other more versatile systems such as IMUs, goniometers, 2-D video-based analysis, among others. This is fundamental due to the differences in accuracy that each of them may have and to the intrinsic registration variability of each specific device. The latter could be affected by instrumentation protocols, the hardware used, the version of the software due to raw data processing, and even environmental aspects. To ensure the validity of using motion capture systems other than photogrammetry to capture the data required to compute the rSCI-GDI, firstly it is necessary to validate the equivalence of the kinematic data in the joints used to compute the index captured with both systems.

In case other centers with gait datasets from other neurological injuries are interested in developing an injury-specific gait deviation index, we encourage them to explore mathematically the reduction of the 9 joints originally considered for the GDI (Schwartz and Rozumalski, 2008) to use only the joints considered relevant for each specific population. Adding other joint movements that are considered relevant can also be explored. By doing so, a more generalizable index could be obtained by focusing on the kinematic movements that characterize the kinematic patterns of each specific population and reducing the variability generated by external factors that are not related to the impairment caused by the injury. Additionally, the rSCI-GDI or an index computed with data of various neurological diseases, could be useful as a feature in characterizing neurodegenerative diseases as a whole, as suggested in (Mengarelli et al., 2022).

## 5 Conclusion

The rSCI-GDI effectively represents gait variability of adults with iSCI as does the SCI-GDI, while providing more generalizable results and equivalent or stronger correlations with clinical tests validated in the population. It can be computed only with gait kinematics of the sagittal planes of hip, knee and ankle and hip abduction/adduction. These kinematics can be reliably gathered with simpler systems than photogrammetry. During the derivation of the improved index, it was demonstrated that pelvic movements, hip rotation, and foot progression angle introduce high variability to the dataset of gait patterns of adult population with iSCI, but they have low clinical relevance to characterize gait kinematics of this population. The rSCI-GDI can be calculated using the 14-feature vectorial basis included in the electronic addendum provided.

## Data Availability

The original contributions presented in the study are included in the article/Supplementary Material, further inquiries can be directed to the corresponding author.
